# Temporal Urinary Metabolomic Profiling in ICU Patients with Critical COVID-19: A Pilot Study Providing Insights into Prognostic Biomarkers via ^1^H-NMR Spectroscopy

**DOI:** 10.3390/cimb48010112

**Published:** 2026-01-21

**Authors:** Emir Matpan, Ahmet Tarik Baykal, Lütfi Telci, Türker Kundak, Mustafa Serteser

**Affiliations:** 1 Department of Medical Biochemistry, School of Medicine, Acibadem Mehmet Ali Aydinlar University, 34752 Istanbul, Turkey; ahmet.baykal@acibadem.edu.tr (A.T.B.); mustafa.serteser@acibadem.edu.tr (M.S.); 2 Acibadem Labmed Clinical Laboratories, 34752 Istanbul, Turkey; 3 Divisions of Anesthesiology and Reanimation, and General Intensive Care, Acibadem International Hospital, 34149 Istanbul, Turkey; lutfi.telci@internationalhospital.com.tr; 4 Division of Internal Medicine, Acibadem International Hospital, 34149 Istanbul, Turkey; turker.kundak@internationalhospital.com.tr

**Keywords:** critical COVID-19, ^1^H-NMR, real-time series analyses, metabolomics, urine

## Abstract

Although the impact of COVID-19, caused by SARS-CoV-2, may appear to have diminished in recent years, the emergence of new variants still continues to cause significant global health and economic challenges. While numerous metabolomic studies have explored serum-based alterations linked to the infection, investigations utilizing urine as a biological matrix remain notably limited. This gap is especially significant given the potential advantages of urine, a non-invasive and easily obtainable biofluid, in clinical settings. In the context of patients in intensive care units (ICUs), temporal monitoring through such non-invasive samples may offer a practical and effective approach for tracking disease progression and tailoring therapeutic interventions. This study retrospectively explored the longitudinal metabolomic alterations in COVID-19 patients admitted to the ICU, stratified into three prognostic outcome groups: healthy discharged (HD), polyneuropathic syndrome (PS), and Exitus. A total of 32 urine samples, collected at four distinct time points per patient during April 2020 and preserved at −80 °C, were analyzed by proton nuclear magnetic resonance (^1^H-NMR) spectroscopy for comprehensive metabolic profiling. Statistical evaluation using two-way ANOVA and ANOVA–Simultaneous Component Analysis (ASCA) identified significant prognostic variations (*p* < 0.05) in the levels of taurine, 3-hydroxyvaleric acid and formic acid. Complementary supervised classification via random forest modeling yielded moderate predictive performance with out-of-bag error rate of 40.6% based on prognostic categories. Particularly, taurine, 3-hydroxyvaleric acid and formic acid levels were highest in the PS group. However, no significant temporal changes were observed for any metabolite in analyses. Additionally, metabolic pathway analysis conducted using the Kyoto Encyclopedia of Genes and Genomes (KEGG) database highlighted the “taurine and hypotaurine metabolism” pathway as the most significantly affected (*p* < 0.05) across prognostic classifications. Harnessing urinary metabolomics, as indicated in our preliminary study, could offer valuable insights into the dynamic metabolic responses of ICU patients, thereby facilitating more personalized and responsive critical care strategies in COVID-19 patients.

## 1. Introduction

Since its emergence, COVID-19, caused by SARS-CoV-2 infection, has led to substantial global morbidity and mortality [1]. Although the prevalence of severe cases has declined over time, the virus continues to generate new variants with diverse clinical presentations ranging from mild disease to life-threatening multisystem involvement requiring ICU admission [2,3]. Given this heterogeneity, accurately stratifying patient prognosis remains challenging, and there is a critical need for tools capable of capturing early biological changes that precede clinical deterioration [4]. Rapid phenoconversion and the dynamic metabolic disturbances it induces further complicate clinical decision-making, underscoring the value of metabolomics for identifying biochemical signatures associated with disease progression [5].

Previous studies have attempted to classify COVID-19 severity and prognosis by examining both clinical outcomes [6,7,8] and underlying biochemical mechanisms [9]. Metabolomic investigations using LC-MS [5], gas chromatography–mass spectrometry (GC-MS) [10], and quantitative nuclear magnetic resonance (NMR) spectroscopy [11] have revealed widespread metabolic perturbations involving energy metabolism, mitochondrial dysfunction, amino acid pathways, lipid metabolism, and immune regulation [12].

Despite growing interest in COVID-19 metabolomics, studies specifically addressing real-time prognostic stratification in critically ill patients, particularly those requiring ICU admission, still remain limited. Personalized metabolic screening approaches capable of capturing prognostic differences based on metabolite alterations in ICU-treated patients are essential but underexplored. Approximately 15–25% of hospitalized COVID-19 patients require intensive care, with mortality rates reaching up to one-third in this subgroup, placing immense pressure on specialized healthcare resources worldwide [13]. The marked heterogeneity among ICU patients, combined with the urgency of initiating appropriate interventions, significantly complicates clinical decision-making regarding therapeutics, supportive care, and resource allocation [14]. In this context, metabolic biomarker monitoring represents a promising strategy for early detection of biological disturbances before clinical deterioration. Non-invasive sample types such as urine offer a practical, patient-friendly alternative to traditional biofluids [15]. Urine permits repeated, low-burden sampling and captures both acute metabolic changes and longer-term physiological alterations, making it a valuable matrix for dynamic disease monitoring and prognostic assessment in critical care settings [16,17].

Previous metabolomic studies have revealed extensive biochemical disturbances associated with SARS-CoV-2 infection, highlighting its broad impact on host metabolism. Alterations in energy-related metabolites such as D-fructose, lactic acid, succinic acid, guanosine monophosphate, citric acid, and 2-palmitoyl-glycerol indicate disrupted cellular energy homeostasis [18]. Key metabolic pathways, including the TCA cycle, purine metabolism, polyamine synthesis, and nicotinamide metabolism, are also affected [19], alongside increases in acylcarnitines and ketone bodies reflecting mitochondrial stress in severe cases [20]. Amino acid metabolism shows similar dysregulation, with changes in glutamine, arginine, histidine, tryptophan, and 3-methylhistidine linked to altered immune responses [5,8,10]. Elevated aromatic amino acids in patients with metabolic comorbidities further suggest impaired hepatic and insulin-related pathways [5,20], while reductions in apolipoproteins A1 and A2 highlight additional metabolic disruption [5]. Activation of the kynurenine pathway—with decreased tryptophan and increased kynurenine and anthranilic acid—is associated with immune imbalance and poor prognosis in ICU patients [21]. Further disturbances include altered methylation-related metabolites and elevations of creatine, 4-hydroxyproline, and gluconic acid, indicating hepatic and renal stress [22], as well as urea-cycle alterations involving arginine, citrulline, and ornithine [21]. Additional shifts in threonine, histidine, lysine, tyrosine, and N,N-dimethylglycine further support the systemic metabolic disarray induced by SARS-CoV-2 infection [23].

Importantly, while most of these insights have been derived from either serum or plasma based analyses, there is a growing recognition of the value of non-invasive matrices such as urine. Urine, being easily obtainable without the need for invasive procedures, offers a practical solution for dynamic disease monitoring especially in ICU settings where patient fragility might limit repeated blood draws. To address the gap in real-time, non-invasive metabolomic monitoring of critically ill COVID-19 patients, this preliminary study utilized urine samples collected from ICU-admitted patients at 4 defined time points during April 2020. By leveraging this longitudinal sampling design, we aimed to capture both acute and chronic systemic metabolic alterations associated with SARS-CoV-2 infection in relation to prognostic differences. Specifically, this preliminary study sought to evaluate real-time changes in urinary metabolite levels and their associations with distinct prognostic trajectories. Furthermore, our preliminary analyses also aimed to explore prognostically and temporally significant urinary metabolites mapped onto specific metabolic pathways, offering insights into the underlying biochemical processes that might reflect COVID-19 severity in the ICU setting.

## 2. Materials and Methods

### 2.1. Research Design and Patient Demographics

In this study, a total of 32 urinary samples were analyzed, retrospectively collected at 4 predetermined time points (Day 0 (10 April), Day 1 (13 April), Day 2 (17 April), and Day 3 (20 April)) during April 2020 and stored at −80 °C until metabolomic profiling via ^1^H-NMR spectroscopy. The study consisted of eight critically ill COVID-19 patients (4 male, 4 female; aged 48–77 years) who had been admitted to the ICU for advanced supportive treatment due to clinical deterioration. These individuals were subsequently stratified into three prognostic categories based on their outcomes: healthy discharged (HD, n = 4), polyneuropathic syndrome (PS, n = 2), and deceased (Exitus, n = 2). The PS group consisted of patients who exhibited clinical features such as distal muscle weakness, muscle atrophy, sensory disturbances including numbness, tingling and burning sensations, diminished or absent deep tendon reflexes, and findings resembling Guillain-Barré syndrome during their ICU stay. Additionally, all participants met the World Health Organization (WHO) criteria for critical illness, presenting with a score of 5–8 on the WHO Ordinal Scale for Clinical Improvement (OSCI), thereby confirming the severity of their clinical condition [24].

All patients included in the study had laboratory-confirmed SARS-CoV-2 infection, verified through reverse transcription quantitative polymerase chain reaction (RT-qPCR) using nasal swab samples (vNAT^®^ Transfer Tube, Bioeksen Molecular Diagnostics), performed with the CFX96 Touch Real-Time PCR Detection System (Bio-Rad, Hercules, CA, USA) and the Bio-Speedy^®^ COVID-19/Flu RT-qPCR Kit (Bioeksen Molecular Diagnostics, Istanbul, Turkey). Prognostic classification was determined based on each patient’s clinical outcome at the end of their ICU stay. For the PS and Exitus groups, selection was constrained by factors such as limited patient availability, sampling feasibility at designated time points, and sufficient sample volume for analysis. Ultimately, 16 urinary samples collected longitudinally from four individuals across four time points, were included for the PS and Exitus prognostic groups. Likewise, the HD group comprised 16 samples from 4 patients, matched for age and sex, with samples obtained at equivalent intervals. Importantly, while the HD group served as a control group in this study, it should not be interpreted as a conventional healthy control group. Instead, these patients were considered representative of critically ill individuals who ultimately recovered and were discharged from the ICU, providing a basis for assessing distinct metabolic responses and trajectory-specific changes among patients with critical COVID-19. Additionally, all of the samples were acquired in the early phase of the pandemic, prior to the widespread administration of vaccines and potential confounding exposures such as different kind of medications. Demographic details of all included patients including age, sex, duration of ICU stay, and prognostic outcome data was presented in Table 1.

This study was conducted according to the guidelines of the Declaration of Helsinki and approved by the Acıbadem Healthcare Institutions Medical Research Ethics Committee (Confirmation number: 2023-15/523), and informed consent was not required for this study because of its retrospective design and the use of previously collected and stored patient urine samples. In addition, patient information was kept confidential, taking patient privacy into consideration.

### 2.2. Sample Preparation for ^1^H-NMR Spectroscopy

Prior to analysis, all samples previously stored at −80 °C were thawed at 4 °C for one hour. After a brief vortexing step to ensure homogeneity, the samples were centrifuged at 14,000× *g* for 5 min at 4 °C. Following centrifugation, the supernatants were carefully separated for subsequent preparation. Each sample was prepared by adding a commercially prepared pH 7.4 sodium phosphate buffer (0.5 M KH_2_PO_4_ and 0.05% TSP in D_2_O; Bruker, Billerica, MA, USA) and urine at a 1:9 ratio, resulting in a final volume of 600 µL for each sample. Prepared mixtures were gently mixed by manual inversion for approximately one minute and subsequently transferred into 5 mm SampleJet™ NMR tubes for spectroscopic analysis [25].

### 2.3. Quantitative ^1^H-NMR Spectroscopy and Sample Processing Workflow

All NMR analyses were conducted using a 600 MHz Bruker Avance IVDr system (Bruker) equipped with a 5 mm BBI probe and an integrated Bruker SampleJet™ robotic cooling unit maintained at 5 °C. The analysis protocols were based on validated methodologies established in previous COVID-19 studies [26], and appropriate system calibrations were completed prior to measurement. For quality control, the B.I. BioBank QC™ module (Bruker) was employed, while metabolite quantification was performed using the B.I. QUANT-UR™ module (Bruker). Total number of 150 metabolites were assessed in urine samples, including methionine, fucose, lysine, hippurate, 2-phenylpropionate, glutamine, 3-indoxyl sulfate, uridine, formic acid, malic acid, pyruvic acid, 3-methylhistidine, trigonelline, taurine, creatinine and many more, covering major biochemical metabolites such as sugars and their derivatives, amino acids and related compounds, keto acids and their derivatives, amines and alcohols, carboxylic and fatty acids, and sulfonates.

Subsequently, one-dimensional (1D) ^1^H-NMR spectra was acquired at 300 K using Bruker’s TopSpin software (version 3.6.2). Standard solvent-suppressed 1D NMR experiments consisted of 32 scans and were completed within a total acquisition time of approximately 4 min [15]. To ensure spectral quality and verify sample integrity, a nuclear Overhauser effect spectroscopy (NOESY) experiment was first performed. Metabolite quantification was subsequently carried out using the B.I. QUANT-UR™ module. To suppress signals from macromolecules, a Carr–Purcell–Meiboom–Gill (CPMG) pulse sequence was applied. In addition, statistical total correlation spectroscopy (STOCSY) was implemented based on prior studies to analyze and confirm characteristic signal patterns for each metabolite [27]. The observed signal patterns related to the metabolites were integrated and correlated to the IVDr measurements to validate the quantification. Furthermore, the NMR metabolites were quantified using the external reference based on an electronic reference for in vivo concentration (ERETIC) calibration [28]. Calibration was integrated using the PULCON (pulse length-based concentration determination) principle, as described in the previous literature [29]. A synthetic reference signal with a known concentration (10 mM) was added to a solvent-suppressed region of the 1D NMR spectrum for external referencing. Final metabolite concentrations were confirmed using a certified reference material aligned with B.I. Methods QC procedures. All sample preparation protocols and NMR methodologies for human biofluids (urine samples) were adapted from the standardized approach described by Dona et al. [30].

### 2.4. Statistical Evaluation and Data Interpretation

All statistical analyses in this study were conducted using online tool ‘MetaboAnalyst 6.0’ (www.metaboanalyst.ca, accessed on 14 November 2024). Missing values in the dataset were handled by referencing the known detection limits specific to each variable. All spectral data were normalized using logarithmic data transformation (base 10) and auto-scaling (mean-centered and divided by the standard deviation of each variable) techniques. Both univariate and multivariate analyses were applied to the dataset. Unsupervised interactive principal component analysis (iPCA) and supervised orthogonal partial least squares discriminant analysis (OPLS-DA) were also performed. These analyses utilized different colors or shapes based on selected metadata and were visualized through 2D and 3D plots. The distribution patterns of the data were assessed using the Shapiro–Wilk test, and group differences were evaluated using the Mann–Whitney U test and the Kruskal–Wallis test, as appropriate.

To evaluate the effects and potential interactions of two independent variables, prognosis and time, a two-way analysis of variance (ANOVA) was conducted, followed by Bonferroni-corrected post hoc tests. To further determine the magnitude of the effect of metabolites that showed statistically significant differences in the two-way ANOVA, linear covariate analyses were also performed. The extent of pairwise differences between the groups were presented in Table 2. Additionally, to identify the primary patterns of variation associated with the two assessed factors and their interaction, ANOVA–simultaneous component analysis (ASCA) was performed. The ASCA was performed depending on previously described algorithms [31], and differences between the compared groups were illustrated using scree plots. Validation of the ASCA was conducted using permutation testing for the factors prognosis, time, and their interaction, respectively. Furthermore, random forest analysis was conducted for both prognosis and time factors. Variable importance plots were generated by evaluating the mean decrease in accuracy, and the influence of relevant analytes on metabolomic classification was subsequently assessed. Metabolites found to be statistically significant (*p* < 0.05) in both two-way ANOVA (F value > 2.5) [32], and ASCA (via leverage/SPE scatter plots) [33] were identified with respect to prognosis, time, and interaction effects. Last but not least, a pathway enrichment analysis based on the Kyoto Encyclopedia of Genes and Genomes (KEGG) database was conducted to explore metabolic pathway alterations among the HD, PS, and Exitus groups. This analysis aimed to identify affected metabolic pathways influenced by metabolites that showed statistically significant differences across groups in the two-way ANOVA and ASCA results. The KEGG findings were interpreted based on the significantly altered metabolites among groups, and pathways were considered affected if they had an impact score > 0.1 and a *p*-value < 0.05.

## 3. Results

### 3.1. H-NMR-Based Metabolic Variations Across HD, PS, and Exitus Groups

To examine metabolic alterations specific to SARS-CoV-2 infection in critically ill COVID-19 patients, 32 urine samples were collected in real time from eight patients treated in the ICU. These patients were classified into three prognostic groups based on their clinical course and outcomes: HD (n = 4), PS (n = 2), and Exitus (n = 2).

Using B.I. QUANT-UR™ analysis, the urinary concentrations of 122 distinct metabolites were quantitatively measured. These included amines and their derivatives, amino acids, carboxylic acids, fatty acids and their derivatives, hydroxy acids such as D-gluconic acid, keto acids, purine, pyrimidine and pyridine derivatives, as well as sugar derivatives. Metabolite concentrations were defined in mmol/L units.

**Figure 1:**
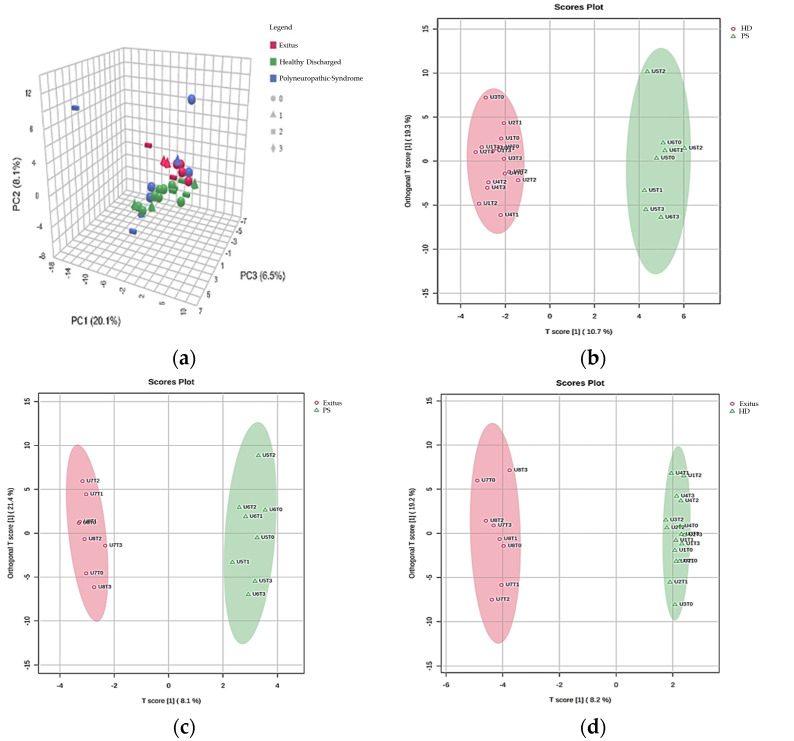
Multivariate analysis of prognostic groups in ICU COVID-19 patients over time. (**a**) A 3D iPCA score plot displays samples differentiated by color (prognostic group) and shape (collection time). Principal components 1, 2, and 3 together account for 34.7% of the total variance, with PC1 explaining the largest proportion. (**b**–**d**) 2D OPLS-DA score plots providing better discrimination between different prognostic groups (HD, PS, and Exitus) according to the collection times of the samples in patients with COVID-19 treated in the ICU.

To distinguish metabolic patterns across prognostic groups and collection times, an interactive principal component analysis (iPCA) was conducted, generating a 3D scores plot. Principal components 1, 2, and 3 together explained 34.7% of the total variance (PC1: 20.1%, PC2: 8.1%, PC3: 6.5%). To further enhance the discrimination between prognostic groups based on sample collection times in ICU-treated COVID-19 patients, two-dimensional OPLS-DA plots were generated. These plots revealed distinct separations between each pair of prognostic groups (Figure 1).

**Figure 2:**
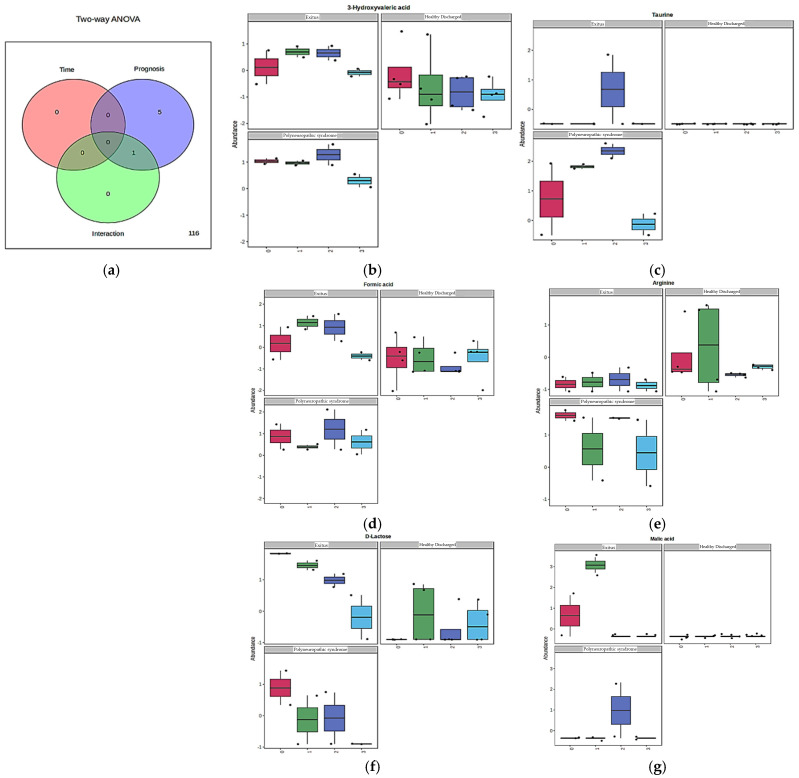
Prognostic and temporal variability of key urinary metabolites in ICU COVID-19 patients. (**a**) A Venn diagram shows the number of metabolites identified as significant by two-way ANOVA based on prognosis, collection time, and their interaction. (**b**–**g**) Box–whisker plots illustrate the relative abundance of taurine, 3-hydroxyvaleric acid, formic acid, arginine, D-lactose and malic acid across prognostic groups (HD, PS, and Exitus) and at each sample collection time point (0, 1st, 2nd and 3rd time points).

Following PCA and OPLS-DA, which revealed clear distinctions between groups, a two-way ANOVA with post hoc Bonferroni correction was performed to identify metabolites contributing to this separation. The analysis assessed the effects of prognosis, sample collection time, and their interaction on group differentiation. Metabolites driving significant separation were identified based on the criteria of F > 2.5 [32] and *p* < 0.05. These thresholds were applied independently to evaluate the impact of each factor separately. It should also be noted that, while the unsupervised PCA did not provide a clear temporal separation of samples, this was expected given the complexity of simultaneously considering prognosis and time as interacting factors. In this context, PCA was primarily presented to illustrate the overall distribution of all evaluated samples in a spatial projection, highlighting the intrinsic overlap and complexity of the dataset when both dimensions were analyzed together. By contrast, more distinct group separations according to the prognosis were evaluated in the OPLS-DA models, whereas the identification of metabolites driving significant inter-group differences was carried out through the two-way ANOVA framework. A summary of the results was provided in Table 3. Specifically, taurine, 3-hydroxyvaleric acid, arginine, formic acid, and D-lactose showed significant differences between groups based solely on the prognosis factor, while malic acid was significantly associated with both prognosis and the interaction factors (Figure 2). No metabolites demonstrated significant differences across time factor alone in the two-way ANOVA. Box–whisker plots illustrated the distribution of metabolites with significant variation based on prognosis and collection time (Figure 2). Additionally, linear covariate analysis was performed to quantify the effect sizes of these metabolites across groups, as identified by two-way ANOVA (Table 2). Analysis of the box–whisker plots and corresponding effect sizes from covariate analysis revealed distinct group-specific metabolite changes in samples. Levels of 3-hydroxyvaleric acid and formic acid were elevated in the PS group compared to the HD group by 1.52 and 1.39 fold, respectively, and to a lesser extent in the Exitus group by 0.97 and 1.07 fold. Taurine levels were significantly higher in the PS group (1.68 fold increase vs. HD), while the Exitus group showed a smaller increase relative to HD (0.29 fold). D-lactose levels exhibited a more pronounced increase in the Exitus group (1.50 fold vs. HD) compared to a modest increase in the PS group (0.43 fold vs. HD). For arginine, a significant elevation was observed in the PS group (1.11 fold vs. HD), whereas the Exitus group showed a significant decrease ((−) 0.69 fold vs. HD). Lastly, although malic acid was found to be significantly associated with both prognosis and interaction factors in the two-way ANOVA, it was not found to be statistically significant in the covariate analysis based on prognosis factor (*p* = 0.21). Therefore, malic acid could not be considered as a reliable discriminator between prognostic groups in this study.

### 3.2. Identification of Prognosis, Time, and Interaction-Based Significant Patterns

**Figure 3:**
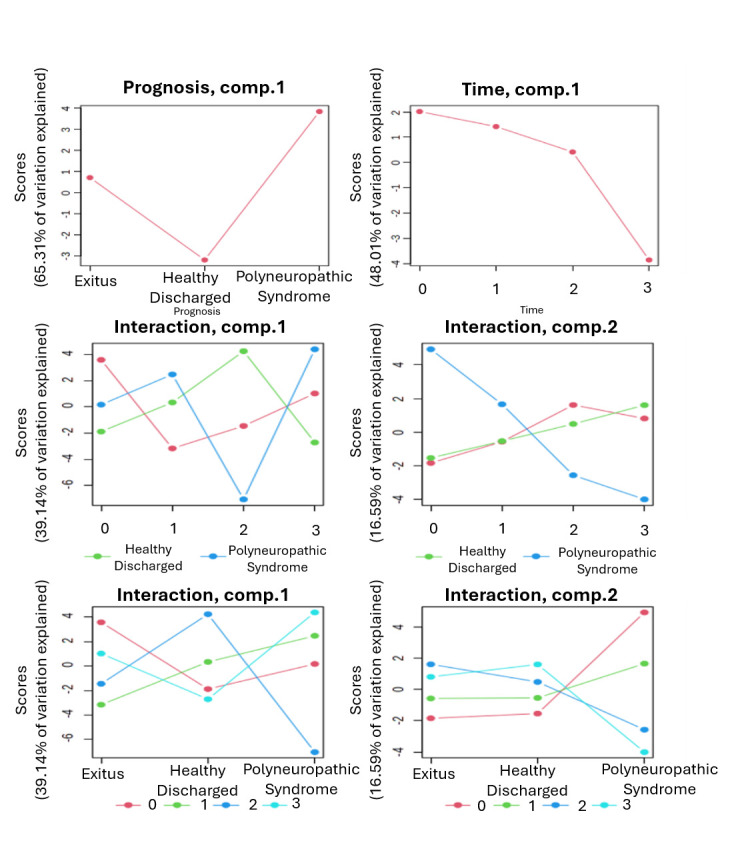
Principal metabolic variation patterns across time and prognostic groups in ICU COVID-19 patients. Scree plots illustrate the major trends in metabolite fluctuations across sample collection time points (0, 1st, 2nd, and 3rd), prognostic categories (HD, PS, and Exitus), and their interaction. The scores indicate the percentage of variance explained by each factor across the groups.

**Figure 4:**
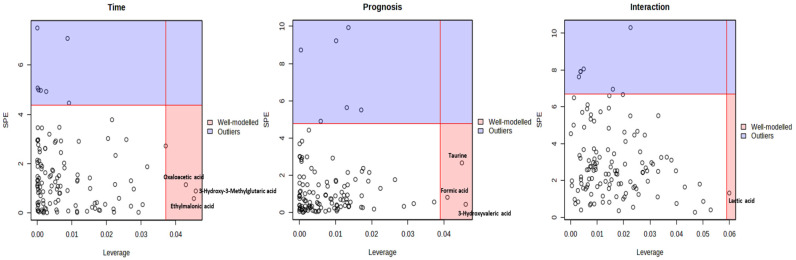
ASCA leverage/SPE analysis identifying significant metabolites associated with time, prognosis, and interaction factors. Metabolites located within the red region exhibit high leverage and low SPE, indicating strong alignment with the major expression patterns captured by the sub-models. In contrast, those in the blue region represent outliers, showing expression patterns that diverge from these trends. Metabolites in the red zone were considered well-modeled and specifically highlighted.

To distinguish prognosis-related metabolite variations from those driven by time, ASCA was applied [34]. This method decomposes the total variation in the dataset into components attributable to individual factors and their interactions [31]. ASCA was used to identify well-modeled components linked to prognosis, collection time and their interaction. Scree plots were employed to visualize the relationship between eigenvalues and factors, revealing significant drops in eigenvalue magnitude and guiding the selection of the number of components to extract (Figure 3). The principal patterns associated with prognosis, collection time, and their interaction were visualized in the score plots based on PC1 of the respective sub-models. Prognosis-related scores were lowest in the HD group, higher in the Exitus group, and highest in the PS group, reflecting increasing levels of explained variance across these prognostic categories. When examining score trends based on collection time, a consistent decrease in variance scores was observed from the initial to the final collection point (Figure 3). Additionally, leverage/squared prediction error (SPE) plots were used to highlight key contributors to each model, with metabolites showing high leverage and low SPE considered significant (Figure 4).

**Figure 5:**
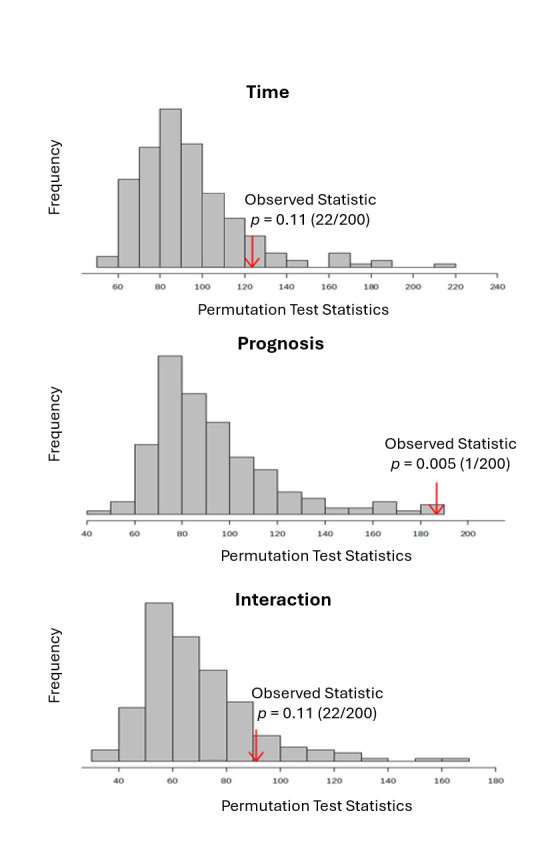
Permutation-based validation of ASCA models. Statistical significance was only observed for prognosis with *p* < 0.05.

Model validation was performed using a permutation test (200 times), confirming the statistical robustness of the ASCA model for prognosis, collection time, and their interaction. Prognosis factor had a significant effect on the urine metabolome (*p* = 0.005), whereas collection time (*p* = 0.11) and the interaction between the two factors (*p* = 0.11) were not statistically significant as shown in Figure 5. These findings also indicated that prognosis and collection time functioned as independent factors, given the absence of a significant interaction. Following model validation, subsequent analysis identified taurine, 3-hydroxyvaleric acid, and formic acid as the metabolites with significant effects on prognosis. Although oxaloacetic acid, 3-hydroxy-3-methylglutaric acid, and ethylmalonic acid appeared to be well-modeled in relation to collection time, and lactic acid was linked to the interaction between time and prognosis, the permutation-based validation showed no statistical significance for either factor. Therefore, these metabolites could not be considered to have a significant impact on the urinary metabolome.

**Figure 6:**
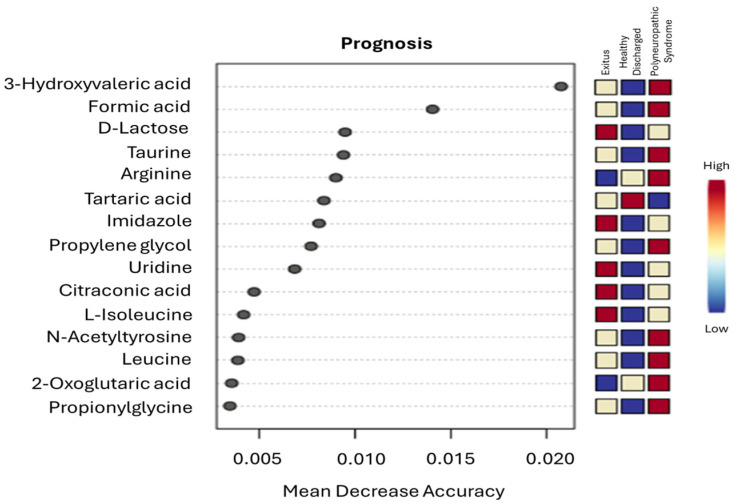
Random forest analysis. Variable importance plot illustrating key prognostic metabolite predictors in ICU COVID-19 patients, ranked by mean decrease in accuracy. Higher values indicate greater importance in distinguishing prognostic groups, with red representing metabolites of higher predictive relevance and blue indicating lower importance in the model evaluation.

To further identify metabolites with the strongest discriminatory power across prognostic groups, a supervised random forest analysis was conducted. Metabolite importance was assessed using the mean decrease in accuracy, where higher values reflected greater contribution to model performance (Figure 6). The random forest model demonstrated moderate prediction of prognosis, with an out-of-bag (OOB) error rate of 40.6%. Among all metabolites, 3-hydroxyvaleric acid and formic acid emerged as the most influential metabolites driving group differentiation.

### 3.3. Metabolic Pathways Exhibiting Significant Alterations

Urine sample datasets from ICU patients, categorized by prognosis, were subjected to pairwise pathway analysis using the KEGG database via MetaboAnalyst (version 6.0). Key metabolic pathways potentially disrupted across groups were identified through pairwise comparisons, based on significant metabolites determined by two-way ANOVA or ASCA. Pathway significance was defined by a *p*-value < 0.05 and an impact score > 0.1. Among the 4 identified KEGG pathways, only 2 (taurine and hypotaurine metabolism, and glyoxylate and dicarboxylate metabolism) met the criteria for significance, primarily due to the impact of taurine and formic acid metabolites, which consistently showed overall statistical significance across all analyses in the study. Specifically, taurine was found to strongly differentiate the PS group from the HD group through its high impact on the ‘taurine and hypotaurine metabolism’ pathway. Similarly, formic acid distinguished the PS and Exitus groups from the HD group via alterations in the ‘glyoxylate and dicarboxylate metabolism’ pathway (Table 4). In contrast, no significant metabolic pathway differences were identified between the PS and Exitus groups based on the associated metabolites.

Finally, KEGG pathway analysis was conducted for metabolites that demonstrated group-level differences either only in the two-way ANOVA (arginine, D-lactose, and malic acid) or only through ASCA (3-hydroxy-3-methylglutaric acid, ethylmalonic acid, oxaloacetic acid, and lactic acid) without passing permutation-based validation tests. While arginine showed some pathway-related differences across prognostic groups, these changes did not show statistical significance and therefore, were not considered reliable within the context of this study.

## 4. Discussion

Although the mortality and morbidity associated with SARS-CoV-2 infection have decreased since the early pandemic, emerging variants continue to pose a risk for severe disease requiring ICU admission, particularly among immunocompromised, elderly, or comorbid individuals [2]. Since the onset of the pandemic, numerous metabolomics studies using serum, plasma, and urine matrices, via ^1^H-NMR and LC/GC-MS, have investigated the biochemical effects of SARS-CoV-2 infection [14,35,36]. However, research simultaneously examining both prognostic differences and real-time metabolic changes in urine from critically ill ICU patients, especially during the pre-vaccination phase, remains limited.

Critically ill COVID-19 patients admitted to the ICU often present with severe respiratory and systemic complications, including pneumonia-like symptoms, ARDS, and multi-organ failure, with mortality rates reported between 40% and 50% [33,37]. Expanding metabolomic research across different biofluids is therefore essential for improving our understanding of disease progression and for supporting more timely and targeted clinical interventions.

In this preliminary study, we investigated urinary metabolite alterations in critically ill COVID-19 patients with differing prognoses using real-time collected urine samples obtained during the initial phase of the pandemic (April 2020). The study aimed to identify metabolite differences and associated metabolic pathways linked to disease severity. Importantly, all samples were collected before widespread vaccination and standardized treatment protocols, reducing confounding therapeutic effects and strengthening the interpretability of the metabolomic findings. By assessing both temporal fluctuations and prognostic trajectories, this study provides early insights into metabolite-level changes that may serve as potential indicators of clinical outcomes in ICU-treated COVID-19 patients.

Significant alterations were identified in the levels of specific metabolites including taurine, 3-hydroxyvaleric acid, formic acid, arginine, malic acid and D-lactose during prognostic evaluation, suggesting that differences in patient prognosis in the ICU might be associated with distinct disruptions in specific metabolic pathways.

Among the metabolites that showed statistically significant differences in urinary levels across the compared prognostic groups, taurine exhibited a slight increase in the Exitus group relative to the HD group, while a more pronounced elevation was observed in the PS group (Table 2). Taurine, a sulfur-containing amino acid derivative, plays a critical role in various biochemical and physiological processes within the body. In the context of ICU-admitted patients with viral infections such as COVID-19, monitoring changes in urinary taurine levels may provide valuable insight into prognostic trajectories and support clinical decision-making during patient management. Taurine is also essential for several key functions, including bile acid conjugation, osmoregulation, membrane stabilization, and antioxidant defense. Additionally, it contributes to the regulation of immune responses, mitochondrial function, and calcium homeostasis [38]. In critically ill COVID-19 patients in the ICU, alterations in urinary taurine levels have been reported in some studies to be associated with metabolic disturbances resulting from systemic inflammation, oxidative stress, and organ dysfunction such as liver or kidney injury [39]. Moreover, taurine’s role in modulating inflammation and regulating mitochondrial function further underscores its importance in severe infections and critical illness. Previous studies have shown that taurine deficiency is associated with increased susceptibility to oxidative damage, reduced immune resilience, and poorer clinical outcomes in critically ill patients [38,40]. Consistent with previous studies, our findings also showed that urinary taurine concentrations, and thus its excretion, were lower in HD patients compared to the other prognostic groups. When comparing the PS and Exitus groups, the higher urinary taurine levels observed in PS patients might be attributed to prolonged ICU stays leading to organ damage, extensive muscle loss, or peripheral nerve injury, as suggested by earlier literature.

Previous metabolomic studies in the literature suggest that elevated serum or plasma taurine levels might reflect an enhanced antioxidant response to oxidative stress induced by cytokine storms, whereas decreased taurine levels might indicate impaired biosynthesis associated with organ dysfunction [41]. The same studies also report that elevated plasma taurine levels observed during the acute phase of COVID-19 remain significantly increased in patients even after a 3 month follow-up, compared to control groups. This sustained elevation has been associated with both acute COVID-19 and post-acute COVID-19 syndrome (PACS) [36,41,42]. Additionally, in our study, KEGG pathway analysis identified the ‘taurine and hypotaurine metabolism’ pathway as significantly altered, particularly between the PS group and the control group, primarily due to differences in urinary taurine levels. This finding aligns with previous literature, where this same pathway was reported among the significantly affected metabolic pathways when comparing plasma levels between COVID-19 patients and healthy controls [41], thereby reinforcing the relevance of our results. Moreover, integrative metabolomic studies utilizing LC-MS, GC-MS, and NMR methodologies have consistently reported elevated serum and plasma taurine levels in patients with severe COVID-19, compared to those with mild symptoms or healthy controls [43]. Regarding our present study, significant increases in urinary taurine levels were detected in the PS and Exitus groups compared to the HD group. The elevations in urinary taurine levels might be associated with alterations in renal filtration, disruptions in gut microbiota or dysregulation of bile acid metabolism, whereas decreases could indicate impairments in glomerular filtration or tubular reabsorption [42]. Considering these points, inclusion of various comorbidity factors related to the intensive care treatment processes of patients in both the Exitus and PS groups in the study could provide a more comprehensive explanation of the results. However, given the well-known critical functions of taurine in cardiac muscle, skeletal muscles, and the brain as well as its significant role in neurological function, the notably higher increases in urinary taurine levels observed in the PS group, characterized by having neurological sequelae and prolonged hospitalization, might be highly significant due to multiple muscle losses and peripheral neuron damage, as previously discussed. Supporting our findings, literature studies have also reported positive correlations between urinary or serum taurine level elevations and COVID-19 disease severity [16,36,42]. However, it is important to note that the urinary taurine differences observed across patient groups may also be caused by the relatively high intra-group variability within the PS cohort. Such variability could be influenced by multiple factors, including heterogeneous clinical trajectories, differences in renal function under critical illness or patient specific responses to systemic inflammation, all of which have been suggested as potential modifiers of urinary metabolite excretion in critically ill populations [44]. Therefore, while our results support and extend previous literature, these findings should be validated in larger cohorts with greater statistical power.

Regarding 3-hydroxyvaleric acid and formic acid, other metabolites found to differ significantly among prognostic groups in the statistical analyses, there are currently very few studies in the literature linking these metabolites to COVID-19. In this context, the preliminary data obtained in our study may serve as a guide for future comprehensive investigations that include these metabolites. Formic acid, a volatile organic acid, plays a critical role in many analyses conducted using the LC-MS/MS method due to its properties. In metabolomic studies utilizing LC-MS/MS method, formic acid is commonly employed as a mobile phase additive to improve chromatographic separation, enhance ionization efficiency, maintain pH balance, and stabilize analytes [45]. However, in our study, since a more sensitive and selective metabolic profiling was aimed using the ^1^H-NMR method, the observed levels and variations in formic acid in urine should not be considered associated with the interference effects that may occur broadly in LC-MS/MS analyses. Formic acid, the primary toxic metabolite of methanol, is produced in the body through its metabolism, first converting methanol to formaldehyde and subsequently to formic acid, which can lead to metabolic acidosis or damage to the optic nerve, potentially resulting in blindness [46]. Recent literature, particularly during the COVID-19 pandemic, has reported an increase in methanol intoxication cases, accompanied by the accumulation of formic acid in the body and a corresponding rise in incidences of intracerebral hemorrhage [47]. Additionally, some other studies have demonstrated a positive correlation between increased urinary formic acid concentrations and the severity of Alzheimer’s disease, along with the accumulation of formaldehyde, a metabolite of formic acid, in the body [48]. These studies particularly emphasize that monitoring changes in urinary formic acid levels may serve as a potential and novel biomarker for the early diagnosis of Alzheimer’s disease. Regarding of our study’s results, significantly elevated urinary formic acid levels were observed in the PS and Exitus groups compared to the HD group, suggesting a possible association with poorer prognostic outcomes during ICU treatment in cases of critical COVID-19. Metabolomic studies investigating serum, plasma, or urinary formic acid levels in the context of COVID-19 have also reported that urinary formic acid may vary with age [36], or that its levels may be influenced by the bacterial flora [16]. These studies also note that urinary formic acid concentrations tend to be higher in COVID-19 patients compared to healthy controls. Similarly, in our study, higher urinary formic acid levels were identified in groups with poorer disease prognoses. Furthermore, the separation of the control group from the PS and Exitus groups based on urinary formic acid levels was associated with the ‘glyoxylate and dicarboxylate metabolism’ pathway in the present study. This distinction may be linked to alterations in the intestinal microbiota of these patient groups, as previous studies have indicated that this pathway is influenced by microbial activity in the human gut [42]. Supporting these earlier findings, our current analysis also revealed elevated urinary formic acid levels in critically ill COVID-19 patients with poor prognoses, suggesting a potential association between disease severity and microbiota-related metabolic changes. Collectively, these results contribute meaningful insights to the existing literature on COVID-19 pathophysiology in ICU patients.

Depending on the results of the analyses, 3-hydroxyvaleric acid levels were found to be elevated in both the PS and Exitus groups compared to the HD group. This metabolite is a straight-chain, unbranched five-carbon fatty acid featuring a hydroxyl group at the third carbon. Similarly to formic acid, it can arise as an intermediate product during the fermentation processes of certain microorganisms within the human microbiota or through various fatty acid metabolic pathways [49]. To date, no studies have directly linked 3-hydroxyvaleric acid to COVID-19. However, given its potential association with protein catabolism or the body’s nutritional status, the observed increase in urinary 3-hydroxyvaleric acid levels in PS and Exitus patients compared to the control group may be an expected finding in critically ill COVID-19 patients and may represent a novel contribution to the literature [9]. Additionally, the greater elevation of this metabolite in the PS group relative to the Exitus group could be attributed to prolonged ICU stays and treatment, or to extensive muscle damage resulting from peripheral neuronal injury in these patients.

Although arginine, D-lactose, and malic acid metabolites were found to be statistically significant across prognostic groups in the two-way ANOVA, they were not identified as significant in the ASCA. Nevertheless, several metabolic studies in the literature have investigated these metabolites, with some linking them to various infections, while others specifically report changes in their serum, plasma, or urine levels in the context of COVID-19. One such study identified arginine among the serum metabolites associated with the onset and progression of COVID-19, highlighting the involvement of ‘arginine biosynthesis’ pathway in compared groups [50]. Regarding the results of our study, urinary arginine levels in the Exitus group were lower than those in the control group, consistent with previous findings. This reduction in urinary arginine levels may reflect impaired T cell function, increased catabolic activity, and immune dysfunction in critically ill COVID-19 patients. Conversely, higher urinary arginine concentrations observed in the PS group compared to the control group may indicate a more sustained immune response due to prolonged ICU stays, resulting in elevated arginine excretion. Another study also reported decreased serum arginine levels in both adult and pediatric COVID-19 patients, suggesting its role in immune defense, protein synthesis, and nutritional metabolic processes [51]. In line with these findings, our study identified the Exitus group as having the lowest urinary arginine concentrations, reinforcing existing literature. Moreover, pathway analyses in multiple metabolomic studies have frequently highlighted the ‘arginine biosynthesis’ and ‘arginine and proline metabolism’ pathways as significantly affected [41]. Similarly, our study revealed that these pathways differed significantly among the prognostic groups based on urinary metabolite data, further supporting and expanding on previous research. Considering the cellular and metabolic significance of D-lactose, it is primarily related to carbohydrate metabolism and energy production. In our study, elevated urinary D-lactose levels were observed in both the PS and Exitus groups, with the highest concentrations detected in the Exitus group when compared to the HD group. However, there are currently no published studies that directly support these findings in the context of COVID-19. Nonetheless, previous research has indicated that during infections and increased inflammatory states, D-lactose and other carbohydrates can influence the gut microbiota. The fermentation of D-lactose is known to produce short-chain fatty acids, which can enhance intestinal barrier function and inhibit the translocation of pathogens into systemic circulation [52]. Additionally, D-lactose may modulate immune responses and contribute to the regulation of pro-inflammatory processes [53]. Considering these mechanisms, the lower urinary excretion of D-lactose observed in the HD group may reflect the preservation of its protective physiological functions. Although no studies to date have directly linked D-lactose with COVID-19, our findings provide preliminary evidence that urinary D-lactose levels may vary according to prognostic status in critically ill COVID-19 patients. The notably higher levels observed in the Exitus group, compared to the PS and HD groups, suggest a potential association between disease severity and altered carbohydrate metabolism, warranting further investigation. Regarding of malic acid, it was the only metabolite found to be statistically significant for both the prognosis and interaction factors in the two-way ANOVA, but it was not considered a reliable marker in present study. This was due to the subsequent linear covariate analysis based on the prognosis factor, which yielded a non-significant statistical result (*p* > 0.05), inconsistent with the previous findings.

In the current study, ASCA based on real-time changes in urinary metabolite concentrations of critically ill COVID-19 patients in the ICU identified oxaloacetic acid, 3-hydroxy-3-methylglutaric acid, and ethylmalonic acid as well-modeled metabolites in relation to the time factor. However, due to the results of the permutation-based validation (*p* = 0.11), these metabolites were not considered statistically significant and thus were not retained as reliable indicators.

This preliminary study also had several limitations. Foremost among them was the relatively small sample size, as samples were collected at regular intervals from ICU patients during the early phase of the COVID-19 pandemic. The sampling strategy accounted for prognostic categorization as well as age and sex similarity, which increased the value of each sample but limited the broader applicability of the findings. Consequently, the generalizability of our results to wider populations remained uncertain. Another key limitation of the study was that the control group consisted of patients infected with COVID-19 who were admitted to the ICU and subsequently discharged after recovery (HD group) rather than healthy individuals because of the constraints in collecting compatible samples. Furthermore, while variables such as age, sex, and ICU stay duration were considered, the limited sample size precluded deeper analysis of their associations with temporal or prognostic variations in urinary metabolite profiles. A final important limitation was the lack of detailed clinical data on patients’ pre-existing conditions, co-morbidities, and treatment regimens. The absence of this information restricted the ability to assess how these factors might have influenced metabolic alterations observed in urine samples. To address these limitations, future research should aim to include larger, more diverse cohorts and incorporate comprehensive clinical profiling including comorbidities, pharmacological interventions, and treatment histories, strengthening the interpretive power of metabolomic analyses in predicting clinical outcomes in COVID-19.

## 5. Conclusions

COVID-19 can induce distinct metabolic alterations in urine samples as with serum and plasma, and detecting such changes in urine, which is a non-invasive and easily collectible biofluid even from critically ill patients, may facilitate faster and more practical patient stratification and management based on prognostic differences. In the current study, elevated urinary levels of taurine, 3-hydroxyvaleric acid, and formic acid metabolites in the PS and Exitus groups, compared to the HD group, suggest that these metabolites may serve as potential prognostic biomarkers, contributing important supportive evidence to the existing literature regarding disease progression in critically ill COVID-19 patients in the ICU. Importantly, all analyses were conducted on samples collected independently of vaccine effects, as they were obtained during the early stages of the pandemic. Although the present study did not identify statistically robust real-time temporal metabolite shifts, such associations might emerge with larger sample sizes, allowing for the identification of time-based biomarkers of disease progression. Among the metabolic pathways most affected across prognostic groups, the ‘taurine and hypotaurine metabolism’ pathway emerged as significant (*p* < 0.05), primarily driven by elevated taurine levels. In addition, differences in formic acid levels across groups highlighted the involvement of other relevant pathways, such as ‘glyoxylate and dicarboxylate metabolism’. Therefore, further validation of these findings in larger-scale studies would be critical for advancing personalized monitoring and therapeutic strategies in the ICU, particularly by leveraging non-invasive sample types like urine to support prognostic assessment in critically ill COVID-19 patients.

## Figures and Tables

**Table 1:** Demographics of the patients included in this study. Age and ICU stay data are presented as mean ± standard deviation.

Prognosis	Number of Patients	Number of Samples	Age (Years)	Sex Distribution	ICU Stay (Days)	Group Description
Healthy Discharged	4	16	61.75 ± 11	2 M/2 F	16.5 ± 3.5	Patients recovered fully and were discharged without major complications.
Polyneuropathic Syndrome	2	8	63 ± 12.73	2 M	102 ± 53.7	Patients developed ICU-acquired polyneuropathy but survived.
Exitus	2	8	70.5 ± 9.2	2 F	24 ± 1.4	Patients who unfortunately died during ICU stay.

**Table 2:** Linear covariate analysis of prognostically relevant urinary metabolites. This table presents the effect sizes of urinary metabolites that demonstrated statistically significant differences among prognostic groups in ICU-admitted COVID-19 patients. Metabolites were selected based on criteria of F-value > 2.5 and *p*-value < 0.05. A single asterisk (*) denotes metabolites identified as significant by both two-way ANOVA and ASCA, while a double asterisk (**) indicates significance in two-way ANOVA only. Positive effect sizes reflect increased metabolite concentrations between groups; negative values represent decreased concentrations.

Metabolites	Exitus vs. Healthy Discharged	Polyneuropathic Syndrome vs. Healthy Discharged	F Value	Adj. *p* Value
Taurine *	0.29	1.68	8.24	0.032
3-Hydroxyvaleric acid *	0.97	1.52	7.22	0.034
Arginine **	(−) 0.69	1.11	7.07	0.035
Formic acid *	1.07	1.39	6.65	0.039
D-Lactose **	1.50	0.43	6.45	0.039

**Table 3:** Prognostic and temporal urine metabolite changes identified by integrated two-way ANOVA and ASCA. This table presents metabolites showing significant differences in abundance (*p* < 0.05) as determined by both two-way ANOVA (F > 2.5) and ASCA (based on significant leverage/SPE ratio). Metabolites are grouped according to their association with prognosis, collection time and interaction factors. Taurine, 3-hydroxyvaleric acid and formic acid (*), identified as significant metabolites in both analyses, are highlighted as key metabolites distinguishing between prognostic groups.

Metabolites	Two-Way ANOVA with Post Hoc Bonferroni Correction						ANOVASimultaneous Component Analysis (ASCA)								
	Prognosis		Time		Interaction		Prognosis			Time			Interaction		
	F Value	*p* Value	F Value	*p* Value	F Value	*p* Value	Leverage	SPE	*p* Value	Leverage	SPE	*p* Value	Leverage	SPE	*p* Value
Taurine *	25.69	**0.001**	4.12	0.59	3.18	0.47	0.045	2.66	**<0.05**						
3-Hydroxyvaleric acid *	9.82	**0.024**	0.94	0.89	0.40	0.93	0.046	0.43	**<0.05**						
Formic acid *	8.72	**0.041**	0.27	0.99	0.83	0.78	0.041	0.81	**<0.05**						
Arginine	10.52	**0.023**	0.54	0.94	0.77	0.79									
D-Lactose	12.76	**0.016**	2.04	0.73	2.28	0.51									
Malic acid	11.21	**0.022**	3.57	0.59	8.74	**0.01**									
3-Hydroxy-3-methylglutaric acid										0.048	0.89	**<0.05**			
Ethylmalonic acid										0.045	0.59	**<0.05**			
Oxaloacetic acid										0.043	1.15	**<0.05**			
Lactic acid													0.06	1.32	**<0.05**

**Table 4:** KEGG pathway analysis of significantly altered metabolic pathways. This table presents KEGG pathway analysis based on metabolites identified as statistically significant (*p* < 0.05) through two-way ANOVA and ASCA, considering the effects of prognosis, sample collection time, and their interaction. However, no significant metabolic pathway alterations were found between the PS and Exitus groups. Therefore, no comparison between these groups was included in the table. Metabolic pathways significantly affected by differences in taurine or formic acid levels across the different prognostic groups were also highlighted by single asterisk.

Pathway Name (KEGG)	Polyneuropathic Syndrome/Healthy Discharged			Exitus/Healthy Discharged		
	*p*-Value	Metabolites	Impact	*p*-Value	Metabolites	Impact
Taurine and hypotaurine metabolism *	<0.001	Taurine	0.43			
Glyoxylate and dicarboxylate metabolism *	0.01	Formic acid	0.11	0.005	Formic acid	0.11
Arginine and proline metabolism	0.013	Arginine	0.24	0.015	Arginine	0.24
Arginine biosynthesis	0.023	Arginine	0.40			

## Data Availability

The raw data supporting the conclusions of this article will be made available by the authors on request.
